# On the way to routine cardiac MRI at 7 Tesla - a pilot study on consecutive 84 examinations

**DOI:** 10.1371/journal.pone.0252797

**Published:** 2021-07-23

**Authors:** Theresa Reiter, David Lohr, Michael Hock, Markus Johannes Ankenbrand, Maria Roxana Stefanescu, Aleksander Kosmala, Mathias Kaspar, Christoph Juchem, Maxim Terekhov, Laura Maria Schreiber

**Affiliations:** 1 Comprehensive Heart Failure Center Wuerzburg (CHFC), Chair of Molecular and Cellular Imaging, Wuerzburg, Germany; 2 Department of Internal Medicine I, Cardiology, University Hospital Wuerzburg, Wuerzburg, Germany; 3 Department of Radiology, University Hospital Wuerzburg, Wuerzburg, Germany; 4 Department of Health Services Research, Carl von Ossietzky University of Oldenburg, Oldenburg, Germany; 5 Departments of Biomedical Engineering and Radiology, Columbia University, New York, New York, United States of America; Faculty of Medical Science - State University of Campinas, BRAZIL

## Abstract

**Introduction:**

Cardiac magnetic resonance (CMR) at ultrahigh field (UHF) offers the potential of high resolution and fast image acquisition. Both technical and physiological challenges associated with CMR at 7T require specific hardware and pulse sequences. This study aimed to assess the current status and existing, publicly available technology regarding the potential of a clinical application of 7T CMR.

**Methods:**

Using a 7T MRI scanner and a commercially available radiofrequency coil, a total of 84 CMR examinations on 72 healthy volunteers (32 males, age 19–70 years, weight 50–103 kg) were obtained. Both electrocardiographic and acoustic triggering were employed. The data were analyzed regarding the diagnostic image quality and the influence of patient and hardware dependent factors. 50 complete short axis stacks and 35 four chamber CINE views were used for left ventricular (LV) and right ventricular (RV), mono-planar LV function, and RV fractional area change (FAC). Twenty-seven data sets included aortic flow measurements that were used to calculate stroke volumes. Subjective acceptance was obtained from all volunteers with a standardized questionnaire.

**Results:**

Functional analysis showed good functions of LV (mean EF 56%), RV (mean EF 59%) and RV FAC (mean FAC 52%). Flow measurements showed congruent results with both ECG and ACT triggering. No significant influence of experimental parameters on the image quality of the LV was detected. Small fractions of 5.4% of LV and 2.5% of RV segments showed a non-diagnostic image quality. The nominal flip angle significantly influenced the RV image quality.

**Conclusion:**

The results demonstrate that already now a commercially available 7T MRI system, without major methods developments, allows for a solid morphological and functional analysis similar to the clinically established CMR routine approach. This opens the door towards combing routine CMR in patients with development of advanced 7T technology.

## Introduction

Magnetic resonance Imaging at ultrahigh field strength (UHF) has demonstrated its capability in brain imaging and in other organ systems [1–4]. Cardiac magnetic resonance (CMR) imaging is a valuable non-invasive tool for both the diagnostic evaluation of patients with cardiac pathologies and for research on cardiovascular disease. Advantages include its unique feature of radiation-free tissue characterization and detailed assessment of myocardial morphology and function. Therefore, it is being used for a variety of diagnostic applications such as evaluation of heart failure, suspected myocardial damage and detection of myocardial scarring, dilatation, and right ventricular pathologies [5–8]. Higher field strengths allow for a higher signal-to-noise ratio (SNR) that enables higher resolution and faster image acquisition [9]. The latter has been used for acceleration of 4D aortic flow measurements. The SNR increased by the factor of 2.2 in comparison with 3.0T [10]). This was put to use for a significant acceleration of the net acquisition time with an optimized approach [11]. First feasibility studies have shown that the increased SNR and resolution can be used to improve spectroscopic analysis of cardiac metabolism by detecting ^31^P in a more localized and defined voxel than at lower field strengths [12–15]. The first diagnostic cardiac studies have demonstrated the potential of high-resolution CMR at 7T in both healthy volunteers and carefully selected patients [16–20]. These works have shown the feasibility of CINE imaging at 7T that allowed the analysis of RV and LV function with results comparable to those obtained at 1.5T [17,18,20]. In the first study focusing on patients, Prothmann et al. showed in 13 subjects with hypertrophic cardiomyopathy that CMR at 7T detects myocardial crypts that were not visible on CMR scans at clinical field strengths. However, larger clinical trials to further elucidate these findings have yet to be performed.

The establishment and execution of CMR at 7T is highly challenging. At UHF, B_0_ and B_1_ field inhomogeneities are larger than at conventional clinical field strengths of 1.5T or 3T. Field strength-dependent variations of tissue relaxation times challenge the generation of T_1_-weighted images and favor T_2_- and T_2_*-weighted images. The susceptibility effects increase proportionally with the field strength. Thus the potential to measure and use innovative and new image contrast techniques like T_2_*- or susceptibility-weighted imaging increases as well [21,22]. Specific hardware and pulse sequences are required that on one hand allow high-resolution imaging, and on the other hand solve technical challenges such as magnetic field inhomogeneities, localized energy disposition, and potential heating effects [23,24]. At UHF, it is difficult to estimate the latter, especially in a more generalized approach covering a wide range of weight and body build [23–27]. Besides these more technical factors, anatomical and physiological factors such as subject-specific anatomy, cardiac and respiratory motion, and increased sensitivity to blood flow effects add high demands on the applied techniques.

Hardware developments as well as sequence development offer solutions that yet have to be tested in a larger group of volunteers and patients and are not readily and equally available to all centers [25,28,29]. Consequently, the progress towards a routine application of 7T CMR still remains slow. The available studies are limited to small numbers of volunteers and patients. A necessary step towards the application of 7T CMR in a larger number of study subjects and patients with specific pathologies is to omit limitations based on individual factors such as weight, age, sex, and body built. The aim of this study was to assess the current status and existing, publicly available technology in a series of 84 subsequent CMR examinations at 7T obtained between 2017 and 2020. We focus on the performance of the commercially available hardware and sequence protocols where we implemented 7T-adapted user-selectable pulse sequence schemes known from 3T. Data were analyzed with regard to the overall image quality, evaluation of functional parameters, and subjective acceptance of these CMR scans.

## Methods

### Hardware

All scans were performed on a latest-generation whole-body 7 T MR system (MAGNETOM^TM^ Terra, Siemens Healthineers, Erlangen, Germany) equipped with full 2^nd^- and partial 3^rd^-order (integrated terms: Z3, Z2X, Z2Y, and Z(X2-Y2)) spherical harmonics (SH) shims. The bore size is 60 cm and the magnet length 270 cm. Maximum gradient strengths of 80 mT/m can be achieved with a slew rate of 200 T/m/s. Currently, for the MAGNETOM Terra system no CE (Conformité Européenne) or FDA (U.S. Food and Drug Administration) certification is available for cardiac MRI.

A dedicated thorax coil with 1Tx/16Rx channels was used for cardiac imaging (MRI Tools, Berlin, Germany) [30]. The coil consists of an anterior and a posterior section. The coil has a dual row (2x4) arrangement of 8 rectangular loop elements in each section. The maximal input power limit in the first level SAR safety mode is 45W. The power to local SAR conversion factor (k-factor) is 0.96. For this coil, the vendor-provided safety concept allows measurements without practical limitations regarding sex and weight (minimal subject weight is specified as 35kg). Two devices were employed for cardiac triggering: a dedicated vector ECG (Siemens Healthineers, Erlangen, Germany) and an acoustic triggering system (EasyACT, MRI Tools, Berlin, Germany).

### Volunteers and preparation

All human subject scans were performed with approval from the local ethics committee (Ethics committee of the University of Wuerzburg, correspondence number 7/17-sc, Amendment “EUFIND”) and written informed consent was obtained from all volunteers prior to the imaging procedure. 72 consecutive volunteers were included, among which 11 received a second cardiac examination at 7T, and one subject an additional third one. These repeated visits were scheduled at least one week up to two years after the initial scan. Altogether, 84 cardiac examinations were obtained. Besides the lack of knowledge on the existence of cardiac disease, other exclusion criteria included active electrical implants such as pacemakers or insulin pumps, ferromagnetic or other metal implants such as surgery clips and orthopedic implants, claustrophobia, and medical causes such as pregnancy.

The group of subjects included 32 male and 40 female volunteers (19–70 years, mean age 30 years). The mean weight was 70 kg (50–103 kg, median 70 kg) and the mean BMI 23.5 kg/m² (17.2–34.5 kg/m², median 23.0 kg/m²), which was slightly less than the mean BMI in a comparable regional population [31].

All volunteers received metal free clothing before entering the scanner room. For CMR scans, two different trigger devices were prepared for each volunteer, the ECG and acoustic trigger systems as described above. The rationale for this approach was to reliably ensure cardiac triggering as a prerequisite for CMR imaging. Prior to the positioning of the volunteers on the CMR table, the volunteers were placed on a stretcher in a supine position. For the ECG lead placement, the respective skin areas were shaved if needed and cleaned thoroughly with a dedicated cleansing paste (Nuprep, Weaver and Company, CO, USA). The positioning of the ECG leads followed the vendor’s recommendation regarding the overall positioning on the left thorax. Leads were placed only in subcostal spaces, and positions directly adjacent to bone structures were avoided. Additionally, the sensor for acoustic triggering was placed at the cardiac apex area [32]. All volunteers were placed in a supine and head-first position on the MRI table and acoustic protection with both earplugs and dedicated headphones were provided. Prior to the positioning within the scanner bore, the quality of the ECG trigger was checked and if needed, skin preparation and lead positioning repeated. After positioning of the volunteer in the scanner, ACT as trigger technique was either chosen as primary trigger technique or as an alternative for the ECG if the latter failed due to the magnetohydrodynamic effect. Throughout the whole imaging procedure, communication with the volunteer was maintained via a two-way speaker system, visual contact and an emergency bulb.

### Imaging protocols

The implemented imaging protocol was set up similarly to the clinical routine at 3T. It contained survey scans in axial, coronal and sagittal orientation. B_0_-shimming was based on the vendor-supplied shim adjustment using a dual gradient-echo sequence. employing the shortest first echo time TE_1_ = 1.02 ms and an inter- echo spacing TE_2_ = 3.06 ms. The shim adjustment volume was planned on the survey scans, covering the whole heart in all three spatial directions. The shim scans were obtained in expiration however, no cardiac trigger was available for the shim scans.

All measurements were performed in first level SAR safety mode. A localizer procedure was defined in order to obtain double-oblique long- and short-axis images of the heart. CINE image data was acquired with a retrospectively triggered T1 weighted spoiled gradient-echo sequence (FLASH). Imaging parameters were set to FOV 340 x 320 mm², slice thickness 6.0 mm, TR 45–74 ms, TE 3.56 ms, flip angle 12–47°, GRAPPA acceleration factor 2 or 3, up to 27 cardiac phases). The GRAPPA acceleration factor was determined by the coil’s g-factor, allowing robust imaging up to a factor of 3. The total scan time was limited to 11–18 sec per slice in order to allow end-expiratory breath-holds. The full short-axis stack covered the complete ventricles with no interslice gap (14–17 slices). Additional CINE long axis measurements were obtained in a four-chamber view (1–5 slices). The aortic flow measurements were performed in 27 examinations using a phase-encoding sequence with a TR of 98.9 ms, TE 3.2 ms, voxel size 1.5x1.5x6.0 mm³, 7 to 9 segments and 20 to 25 phases. The velocity encoding (VENC) was set to 150 cm/s. For cardiac gating of CINE sequences and flow measurements, either ECG or ACT was chosen for each subject based on the signal quality and stability of triggering signal observed using the scanner software. In 15 volunteers, both equally correct working ECG and ACT triggering were used for data acquisition for direct performance comparison. The reference voltage for the RF-pulses was set using the vendor-integrated B_1_-calibration procedure.

### Data and image analysis

The positioning of the volunteer relative to the coils was evaluated regarding its influence on the image quality. On each data set, three retrospective measurements were performed. On the axial and sagittal scout images, the thoracic regions adjacent to the anterior and posterior coil sections with high signal intensity were identified. The measurements were used to assess the distance between these areas with high signal intensity and the sternum. The measurements were performed as following:

- the distance between the upper sternal edge and the lower border of the anterior signal area,
- the distance between the lateral sternal edge and the right lateral border of the anterior signal area, and
- the difference in the anterior and posterior upper border of the signal area.

Cardiac scanning requires shimming within the thoracic cavity containing a variety of tissues with different magnetic susceptibilities as well as bone and air. To analyze the suitability of the vendor-provided partial third-order shim system for cardiac scanning, B_0_ shim currents for 1^st^-, 2^nd^- and 3^rd^-order SH terms were obtained retrospectively from the vendor’s scan protocol and extracted from the DICOM image headers. The complete data set was analyzed regarding the deviation of an individual value of more than two standard deviations from the group’s average and maximum value due to hardware limitations.

Overall image quality and tissue contrast were analyzed with qualitative scores from 1 to 4 for each of the three parameters artifact burden, noise, and overall quality [33]. The scores were defined as following: 1 optimal imaging result, 2 only little disturbance with no consequence for image analysis, 3 notable disturbance that might lead to misinterpretation of the image, 4 diagnostic evaluation of the image is not possible. The score was applied on full short-axis stacks in a segment-specific fashion, with 17 segments in the left ventricle according to the model of the American Heart Association (AHA), and seven segments in the right ventricle [34]. Each segment was rated with the sum of all three parameters, with a score of three being the best possible and a score of 12 being the worst possible score. The overall image quality was correlated with sex, weight, and BMI. The quality scores from the left and right ventricle were separately correlated to the coil positioning, the B_0_ shim currents, the chosen trigger technique and the nominal flip angle.

Functional image analysis included left ventricular (LV) and right ventricular (RV) volumetry on the short axis stacks. In all slices, the end-diastolic and end-systolic endomyocardial borders for both left and right ventricle were manually traced. For the volumetric analysis, trabecula and papillary muscles were considered part of the intraventricular cavity. The outflow tracts were excluded for the volumetric analysis. The stroke volume (SV) was calculated as the difference between the end-diastolic volume (EDV) and the end-systolic volume (ESV). The ejection fraction (EF) and cardiac output (CO) were computed from these measurements as well and standardization to the body surface area (BSA) was calculated. The four-chamber view was used for monoplanar measurements of the LV function. After manually tracing the endomyocardial borders the LV end-diastolic and end-systolic volumes were estimated. The RV fractional area change (FAC) was determined in a similar way by manually tracing the RV endomyocardial borders on the four-chamber view in both end-diastole and end-systole. Additionally, area measurements of the atria on the four-chamber view were included in the analysis. The aortic contour was semi-automatically traced on the phase contrast images for the flow measurements. All image analyses were performed by experienced physicians (TR, AK) using the software Medis Suite MR 3.2 (Medis Medical Imaging Systems, Leiden, The Netherlands).

### Subjects’ acceptance

The subjects’ acceptance of CMR at 7T was analyzed according to a standardized questionnaire based on previously published works [35,36]. The questionnaire was answered by the volunteers immediately after the examination. Inquired aspects included overall (dis-)comfort, the sensation of narrowness within the scanner bore, noise, duration of the examination, vertigo, nausea, visual effects, pain, and peripheral nerve sensation. A scale from 0 (no discomfort at all) up to 10 (utmost uncomfortable) was applied. The volunteers had the chance to include individual remarks in an open section.

### Statistical analysis

The statistical analysis of all data was performed with the software R (Version 3.6.1, [37]). All data were tested for normality with the Shapiro Wilk test. Data was found not to be normally distributed for the segmental image quality, the correlation of sex and image quality, the influence of the chosen triggering method, the influence of the chosen trigger on flow and volumetric measurements, and the comparison of the subjective acceptance during brain and cardiac scans. Therefore, Wilcoxon rank tests with continuity correction were used for their evaluation. The LV and RV functional results were correlated using Pearson’s product-moment correlation. Kendall’s rank correlation was used for the correlation of image quality with the flip angle, the coil position, and the body weight/BMI. The Kruskal-Wallis rank sum test was used for the correlation of the image quality with the B_0_ shim currents. The analysis of the 4-chamber views used Friedman’s ANOVA testing.

## Results

All volunteers but one finished the examination, the latter developed claustrophobia during positioning in the scanner and terminated the procedure prior to the actual scanning. No severe adverse events were detected in any of the 72 subjects participating in this study.

**Fig 1:**
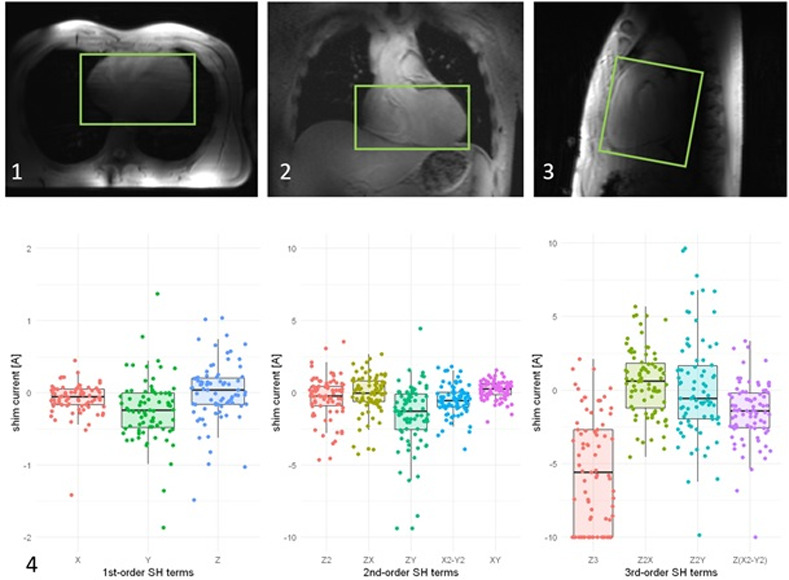
Upper row: The survey scan shows an exemplary shim volume in a transversal (A), coronal (B) and sagittal (C) view. Lower row: Overview over all extracted shim currents for the 1^st^- to 3^rd^-order spherical harmonics (SH) terms. Each color represents the current required to generate a specific SH term. For each current, the minimum and maximum, first and third quartile and the median value are shown. Note that the current required to generate the Z3 component reached the hardware limitation of ±10A for a number of subjects.

The reference voltage for the RF-pulses was found stable across all scanned volunteers when employing the identical RF hardware (420 ± 20V). The results of all B_0_ shim currents of 1^st^ to 3^rd^ order are given in Fig 1. The data show a similar distribution of shim currents in the majority of examinations. However, a small group showed negative saturation in the Z3 term. We interrogated a possible influence of different distributions of shim currents on image quality. There was no statistically significant connection between the image quality and the distribution of the applied currents detected (p = 0.12, df = 2). After the exclusion of data sets which were negatively saturated in the Z3 term, the correlation between image quality and the applied shim currents remained non-significant (left ventricle: p = 0.43; right ventricle p = 0.93), i.e. no significant image degradation was observable despite reaching the upper limit of the Z3 shim component.

### Diagnostic image quality

Among all examinations, 50 included a full CINE short axis stack. The remaining scans either included only a limited number of slices in a short axis orientation, or the chosen number of detected cardiac phases were too low for functional analysis.

**Fig 2:**
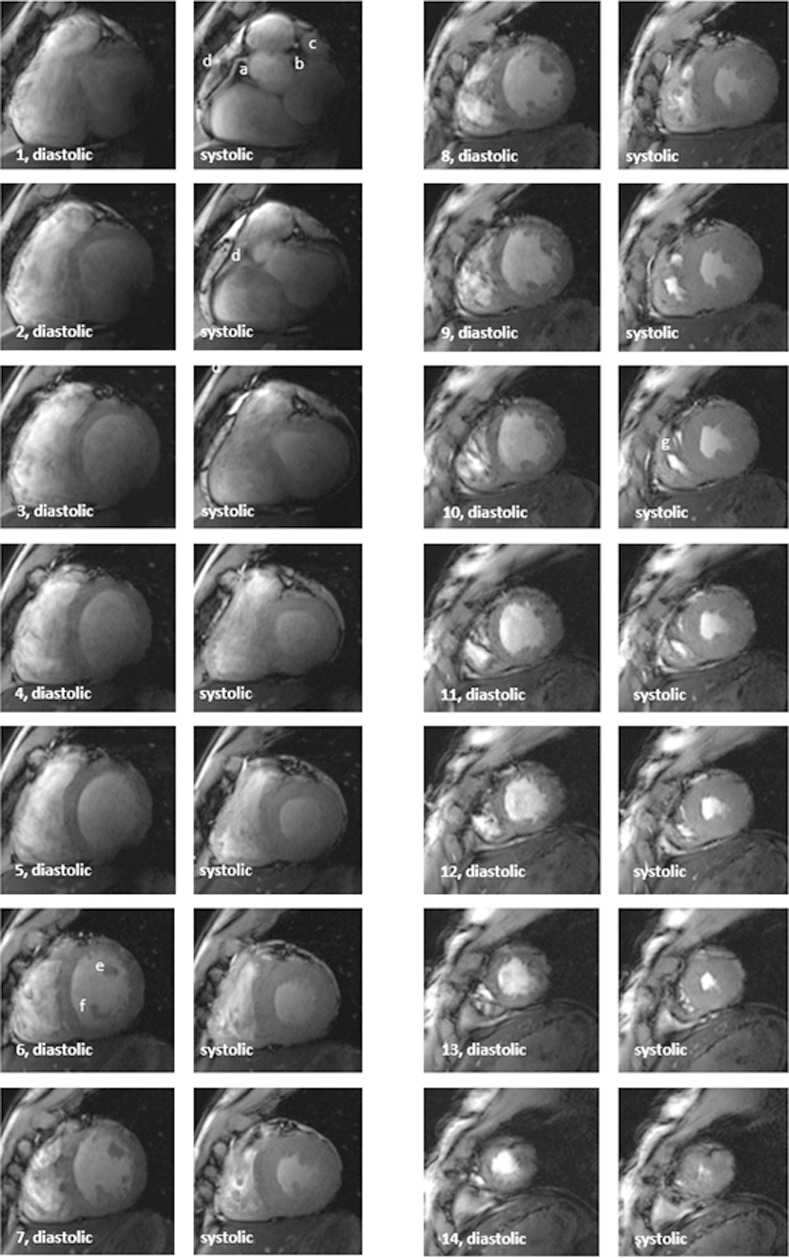
Example of a short axis stack covering the ventricles from the basal slice (image pair 1) to the apical slice (image pair 14). The image quality was rated with 6 (artifact burden 2, noise 2, overall quality 2) in each segment with the exception of segments 4 and 5 (left ventricular basal inferolateral wall), here the overall quality was less and the score rated as 7. All slices were obtained with ACT triggering. Each of the slices is represented pairwise as diastolic and systolic. The right ventricular morphology is detectable throughout all slices, whereas in comparison the basal lateral segments of the left ventricle show a lower contrast. a) proximal segment of the right coronary artery. b) proximal segment of the left main coronary artery. c) left atrial appendaged) right atrial appendage. e) anterolateral papillary muscle. f) posterior papillary muscle. g) moderator band.

The fifty complete CINE short-axis stacks were analyzed regarding image quality (Fig 2). Detected image artifacts predominantly were predominantly caused by motion, flow and aliasing. Flow-related artifacts appear more pronounced during systole than during diastole.

**Fig 3:**
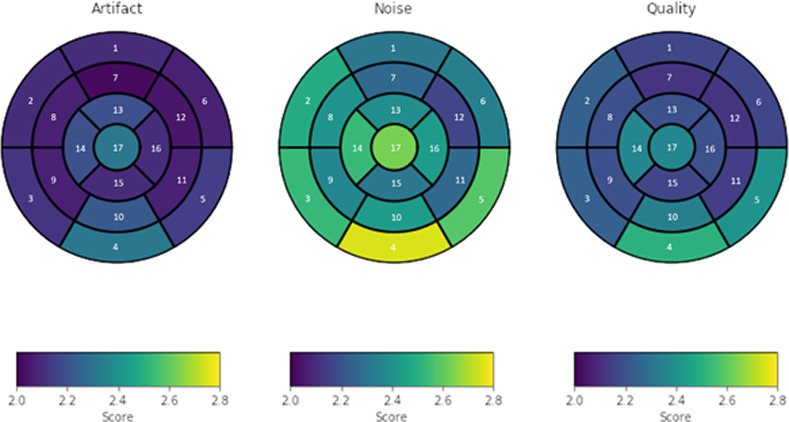
Left ventricular image quality of all 17 left ventricular segments. Representation of each quality parameter, artifact burden, noise, and overall quality as “bull’s eye”. The white numbers indicate the segment number. For each of these parameters, a score from 1 up to 4 was given.

Among all left ventricular segments, 17 (2%) were considered not interpretable based on a rating of 4 points in one parameter, and 29 segments (3.4%) were not interpretable based on a rating of 4 points for at least two parameters (Table 1). Two data sets were excluded from further analysis due to the severely reduced image quality in all segments. The left ventricular basal inferior, inferolateral and anterolateral segments showed a significantly lower image quality compared to the basal septal and anterior segments, with a reduced overall image quality and more pronounced noise (p < 0.001, V = 42) (Fig 3). Contrary to these findings no significant differences were observed between the mid-ventricular segments (p = 0.23; V = 150.5). No significant correlation of left ventricular image quality was observed with body weight (p = 0.06, z = -1.921, BMI (p = 0.1000, z = -1.643), and sex (p = 0.47, w = 358)), respectively. Furthermore, the coil positioning did not significantly influence left ventricular image quality (Table 2).

**Table 1:** Overview over the image quality score in all analyzed left and right ventricular segments.

**Left Ventricle**					
Seg 1	7.23 (6–11; 6)	Seg 7	7.13 (6–10;6)	Seg 13	7.40 (5–12;6)
Seg 2	7.45 (5–11;6)	Seg 8	7.33 (6–10;6)	Seg 14	7.68 (5–12;6)
Seg 3	7.55 (6–11;6)	Seg 9	7.28 (6–10;6)	Seg 15	7.25 (5–12;6)
Seg 4	8.38 (6–11;7)	Seg 10	7.98 (6–11;6)	Seg 16	7.35 (5–12;6)
Seg 5	8.20 (6–11;7)	Seg 11	7.40 (6.11;6)	Seg 17	7,95 (5–12;7)
Seg 6	7.60 (6–11;6)	Seg 12	7.30 (6–11;6)		
**Right Ventricle**					
Seg R1	8.33 (4–10;7)	Seg R4	8.15 (4–10;7)	Seg R7	8.40 (4–12;7)
Seg R2	8.15 (4–10;7)	Seg R5	8.00 (4–10;7)		
Seg R3	8.2 (4–10;7)	Seg R6	7.88 (4–10;7)		

Given are the average values and in brackets the minimum, maximum and median are listed.

**Table 2:** Kendall’s rank correlation of the coil position with the image quality of the left and right ventricle.

	Left ventricle	Right ventricle
**Upper Sternum- lower edge**	p- value 0.57 z = -0.570	p- value 0.09 z = -1.714
**Lateral Sternum- lateral border**	p- value 0.07 z = -2.245	p- value 0.02 z = -2.244
**Offset between anterior and posterior coil element**	p- value 0.60 z = -0.523	p- value 0.07 z = 1.833

**Fig 4:**
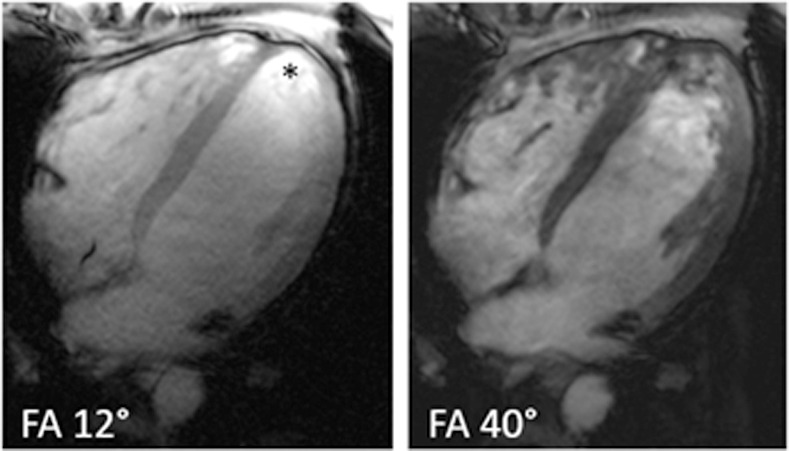
Image quality in a single volunteer depending on the chosen flip angle. Left a flip angle of 12° was chosen, right a flip angle of 40° was used. The image contrast between the myocardium and the blood volume is better on the right side, however, the trabecula and myocardial borders appear more blurred. With a flip angle of 12°, an apical signal overshoot is visible (Asterisk). The overall score for the right ventricle was rated with a score of 7.33 for the diastolic and a score of 7.26 for the systolic phase. The left ventricle was rated with 6.71 for the diastolic and 7.24 for the systolic phase.

Data obtained with ECG triggering showed an overall better image quality than those that were obtained with ACT triggering (ECG triggering: 27 data sets, ACT triggering: 23 data sets, p = 0.05, W = 416). The tissue-to-blood contrast decreased with lower flip angles, with 12° being the lowest and 47° being the highest analyzed flip angle. However, the chosen nominal flip angle did not significantly influence the left ventricular image quality (p = 0.59, z = 0.534) (Fig 4).

The analysis of all right ventricular segments showed that only nine of them were of reduced image quality that would not allow a reliable interpretation (2,5%) (Table 1). No differences were observed between the image quality of the basal and the midventricular slice (p = 0.30; V = 133). The trigger technique did not significantly influence the right ventricular image quality (p = 0.31, V = 133).

Contrary to the LV, the image quality of the RV was significantly dependent on the chosen nominal flip angle. When obtained with lower nominal flip angles, the right ventricular myocardial borders showed a reduced signal overshoot and a better contrast between blood and myocardium (contrast blood-myocardium: p < 0.0001, z = 4.007). The image quality was significantly influenced by the lateral positioning of the anterior cardiac coil (p = 0.03, z = 0.231). Otherwise, the coil position did not significantly influence the RV image quality, but p-values tended to be lower compared to those for the LV (Table 2). Similar to the LV no statistically significant correlations were detected for patient dependent factors (weight: p = 0.156, z = -1.410; BMI: p = 0.238, z = -1.181).

**Fig 5:**
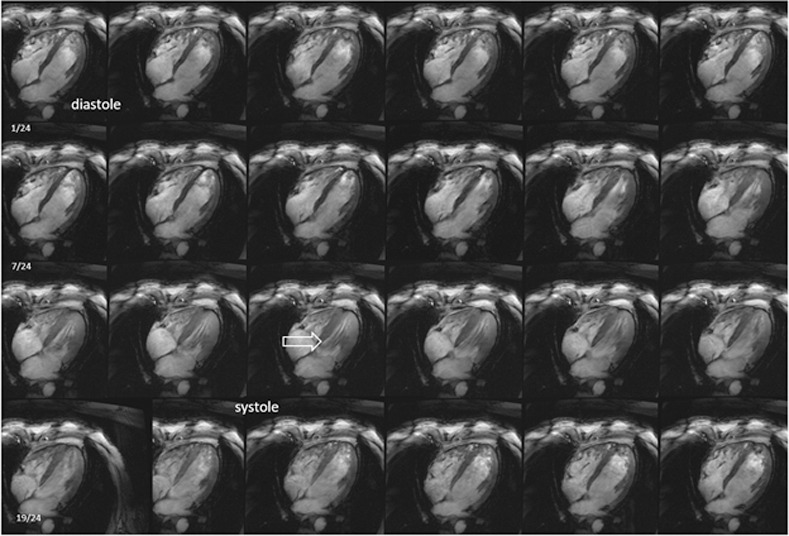
Representative four-chamber view throughout the cardiac cycle, showing 24 cardiac phases beginning and ending with diastole. During systole, prominent flow artifacts in the intracavitary blood volume are detected (white arrow).

The image quality of the four-chamber view was evaluated in a similar way on 35 data sets (Fig 5). The statistical analysis revealed no significant differences between both the ventricles and between the end-diastolic and end-systolic phase (Friedman chi-squared = 7.575, df = 3, p = 0.05566).

### Functional analysis

**Fig 6:**
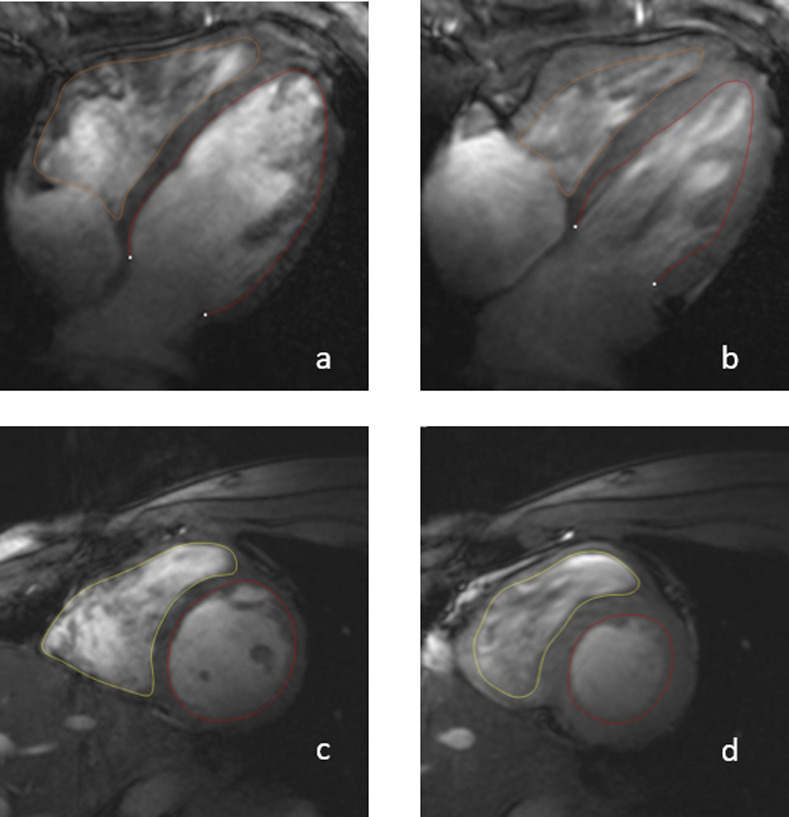
Representative segmentation on four chamber view in a) diastole and b) systole and short axis in c) diastole and d) systole. The segmentation lines were drawn with Medis Suite MR 3.2.

**Fig 7:**
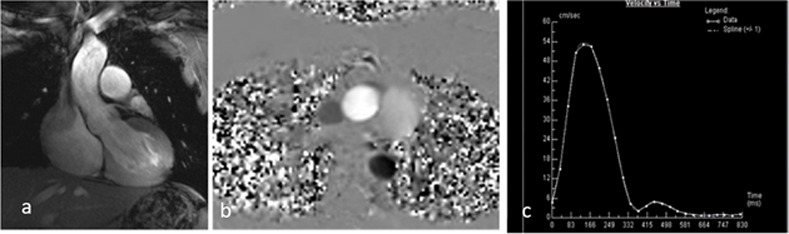
Flow measurements. a) Left ventricular outflow tract, and b) phase encoded view of the aorta. c) Flow curve derived from the flow measurements. The x-axis shows the time in ms, the y-axis the measured aortic flow in ml/s.

The volumetric analysis of the short axis data sets resulted in an overall LV EF of 56% (45% - 66%) and RV EF of 59% (39–72%) (Fig 6). The left and right ventricular end-diastolic, end-systolic, and stroke volume were each indexed to the body surface. In comparison with the volumetric analysis of the short axis, the volumes derived from the long axis were overall smaller (Table 3). The mean left atrial area was 21.8 cm² and the mean right atrial area was 21.1 cm² on the four-chamber view. The FAC as a surrogate marker for the right ventricular function determined mean FAC of 52% (41–70%) in all included healthy volunteers. The flow-derived LV stroke volume did not differ significantly from the short axis volumetric stroke volume (88 ml vs. 87 ml; p = 0.25, v = 237) (Fig 7). No significant intra-individual differences between ECG triggered and ACT triggered flow measurements were detected (p = 0.30, v = 40) (Table 4).

**Table 3:** Functional assessment of left and right ventricular function in healthy volunteers.

	LV (SIMPSON)	RV (SIMPSON)	LV (4CH MONOPLAN)
**EDV**	160 ml (105–242 ml)	150 ml (95–234 ml)	146 ml (95–212 ml)
**ESV**	71 ml (40–115 ml)	62 ml (30–104 ml)	54 ml (24–89 ml)
**SV**	88 ml (37–135 ml)	88 ml (37–140 ml)	92 ml (62–134 ml)
**EF**	56% (45–66%)	59% (39–72%)	63 5% (42–75%)
**CO**	5.7 l/min (4.2–7.8 ml)	5.5 l/min (3.3–8.1 l/min)	5.5 l/min (3.9–7.3 l/min)
**EDV/BSA**	85 ml/m² (60–119 ml/m²)	80 ml/m² (55–108 ml/m²)	78.1 ml/m² (55–105 ml/m²)
**ESV/BSA**	37 ml/m² (23–54 ml/m²)	33 ml/m² (16–54 ml/m²)	29 ml/m² (14–46 ml/m²)
**SI**	48 ml/m² (35–66 ml/m²)	47 ml/m² (21–64 ml/m²)	49 ml/m² (32–68 ml/m²)
**CI**	3.1 l/min/m² (2.2–4.0 l/min/m²)	3.0 l/min/m² (1.9–4.2 l/min/m²)	3.0 l/min/m² (2.1–4.1 l/min/m²)

EDV end-diastolic volume. ESV end-systolic volume. SV stroke volume. EF ejection fraction. CO cardiac output. EDV/BSA end-diastolic volume indexed to the body surface area. ESV/BSA end-systolic volume indexed to the body surface area. SI stroke index. CI cardiac index.

**Table 4:** Flow measurements with ECG and ACT triggering.

	STROKE VOLUME	FORWARD FLOW	BACKWARD FLOW	REGURGITANT FRACTION
**SV (FLOW, ECG)**	92 ml (63–149 ml)	92 ml (63–149 ml)	0	0
**SV (FLOW, ACT)**	88 ml (64–137 ml)	88 ml (64–137 ml)	0	0
**SV (VOLUMETRY)**	88 ml (37–135 ml)	-	-	-

SV Stroke volume.

### Subjects’ acceptance

The questionnaire-based inquiry of subjects’ acceptance of CMR at 7T yielded that the overall acceptance was good (Table 5). Paresthesia most often occurred in the arms (12%), in the abdomen (11%), the legs (10%) and the hands (9%). Other, by far less mentioned areas affected by paresthesia during the examination were the thorax, head, shoulders, feet and fingers. All but two volunteers were willing to return for another examination. Two volunteers reported a feeling of dizziness and fatigue during a prolonged period of time after already having left the research site, one of them being susceptible to motion sickness. In both cases the examination duration did not exceed 60 min. Other written feedback focused on the partially uncomfortable positioning and acoustic noise protection devices as well as the narrowness of the scanner bore. The breath-hold duration during the cardiac examinations were deemed either too long or instructed unclearly by nine volunteers. The temperature in the scanner room was considered tolerable with additional blankets.

**Table 5:** Subjects’ acceptance of brain and cardiac scans at ultra- high field MRI.

SIDE EFFECT	CARDIAC SCANS
**DISCOMFORT**	1.91 (0–6;2)
**DURATION**	1.56 (0–8;1)
**NARROWNESS**	2.00 (0–8;2)
**NOISE**	2.94 (0–8;3)
**PARESTHESIA**	1.81 (0–6;0)
**VERTIGO**	
• **IN** • **DURING** • **OUT**	1.82 (0–8;0) 0.29 (0–6;0) 1.29 (0–8;0)
**NAUSEA**	
• **IN** • **DURING** • **OUT**	0.20 (0–7;0) 0.11 (0–5;0) 0.15 (0–6;0)
**FLASHES**	
• **IN** • **DURING** • **OUT**	0.01 (0–1;0) 0.22 (0–5;0) 0.13 (0–6;0)
**PAIN**	
• **IN** • **DURING** • **OUT**	0.01 (0–1;0) 0.43 (0–7;0) 0.26 (0–7;0)

## Discussion

This manuscript presents the largest number of subsequent cardiac examinations in volunteers at UHF-MRI using latest generation 7T MRI scanner technology and existing pulse sequences and sequence protocols. The data were obtained during the process of establishing the 7T MAGNETOM Terra system at our institution, thus accounting for variable and changing protocols with different research foci at a time. As a result, not all obtained data sets included a full short axis stack and four chamber view.

The approach for this study differs from other previously published works that used individualized setups and customized coil systems. Technical advances such as parallel transmit RF-excitation have been established for brain and musculoskeletal imaging but are not readily and equally available for CMR [38,39]. Currently, no safety concept for parallel transmit imaging using thorax coils is implemented on the scanners, thus requiring modelling of SAR distribution based on both the coil design and the shape and tissue qualities of the individual human body [40–42]. On a smaller scale, individualized set ups for best possible results are feasible, but are not applicable for larger scaled studies [17,18,20].

Our results on 84 subsequent CMR examinations demonstrate that cardiac applications with commercially available setups allow for a solid morphological and functional analysis as foundation for any clinically orientated study and understanding of the cardiac morphology at 7T MRI. With the currently available setup and pulse sequences it is possible to obtain cardiac image data with sufficient image quality that are suitable for analysis of clinically relevant parameters such as left ventricular function (e.g., ejection fraction) as well as flow measurements in the ascending aorta. Nevertheless, a few challenges had to be solved, in particular triggering, setting up a localization scheme as part of the subject preparation, and adjusting pulse sequence parameters for application in cardiac MRI at 7T.

Overall, the results of the volumetric analysis of the short axis data are in line with previously published series at 7T [18,20], demonstrating field-strength-independence of functional cardiac imaging at clinically relevant field strength. In this study, intra-individual comparison between results at different field strengths were not obtained. Reference values are published but require careful interpretation regarding the role of the papillary muscle as either part of the myocardial mass or the blood volume. The reference study of Petersen et al. was performed at 1.5T and considered the papillary muscle as part of the blood volume. In this reference study, the LV function in males ranged between 48 and 69% for males and between 51 und 70% for females. The RV function in males ranged between 45 and 65% and between 47 and 68% in females [43]. In our study, the range was between 45 and 66% for the LV and 47 and 68% for the RV. In general, our results are in line with the reference values. In some of the presented cases, the functional analysis had resulted in reduced ejection fractions, which may be attributed to suboptimal slice planning and a low number of obtained cardiac phases. It is important to highlight, as has been discussed elsewhere, that the functional parameters such as the volumes especially of the RV are dependent on chosen sequences and sequence settings, as well as on the imaging planes used for the analysis, and an uncritical comparison with clinical CMR data should be avoided [44].

The long axis view was more affected by flow artifacts when compared to the short axis view. The blood flow during systole is more orientated in-plane than through-plane, causing prominent artifacts during cardiac phases with high velocity of blood. Nevertheless, the image quality allowed mono-planar measurements of both ventricles. With respect to clinical application, the fractional area change (FAC) of the right ventricle is of particular interest. This has been demonstrated recently in a CMR study at a clinical field strength, where the FAC was found to be a reliable estimate for the right ventricular function. In pulmonary hypertension, FAC reliably predicted impaired right ventricular function, using a FAC value of 35% as cut-off for an impaired RV function [45–47]. Though a short- axis based volumetry is considered the gold standard, the FAC is easier to obtain than performing the complex analysis of the geometrically irregular right ventricular short axis. Additional studies regarding the correlation of echocardiographic FAC and MRI- based FAC would be useful to evaluate this approach further.

One of the key technical challenges in our study was stable cardiac triggering. The magnetohydrodynamic effect increases with field strength, causing distortions of the ECG and, thus, results in the unreliable detection of the QRS-complex generally used for ECG-triggering [32]. ECG placement thus requires meticulous preparation of the study subject, and similarly to clinical scenarios at lower field strengths, repositioning of the leads can be required. At 7T, as an alternative, acoustic triggering has been successfully used [32]. Prior studies demonstrated that ECG and acoustic triggering are interchangeable for volumetric analyses [16,32,48,49]. Adding to these findings, our data show that flow measurements are feasible with both triggering techniques and provide comparable results. In contrast, improved CINE image quality could be obtained with ECG triggered data than with ACT-trigger for the LV but not for the RV. Overall, considering the absolute number of analyzed and excluded cardiac segments, both triggering techniques appear legitimately equal with regard to image quality and flow measurement results.

A technical prerequisite for clinical CMR is adequate B_0_ shimming. The analysis of the vendor-supplied shim adjustments and required currents shows that the provided hardware and adjustment workflows are sufficient to obtain diagnostically usable CINE scans, even though no cardiac triggering was provided. Factors such as sex, weight, and BMI are accounted for by the B_0_ shimming routine and, therefore do not significantly influence the imaging result. When using gradient echo sequences, an increase in the spatial resolution is inversely proportional to the product of readout gradient and TE time. The gradient strength is limited by hardware and safety aspects. Thus, the reduction of the pixel size is obtained by increasing the TE time. However, for TE values above 3 ms, prominent B0 artifacts occur at the interface of myocardial and pulmonary tissue manifested as signal reduction. With the T_2_* time shortened to 4–5 ms) in these regions the use of TE≥3ms causes a k-space filtering effect leading to image blurring. These two factors restrict pushing up limits of spatial resolution of 7T, if no special measures are taken for improvement of B_0_-shimming. In general, flow imaging based on phase mapping is potentially more sensitive than CINE imaging to B0 inhomogeneities. In practice, the aorta has a small cross-section area compared to the myocardium and, thus, for the 1D flow encoding, the B0 heterogeneity can be well managed with appropriate B0-shimming. However, data from our group showed that triggered B_0_ mapping offers superior results and thus would be desirable for future studies [50].

There are two reasons for the wide range of flip angles used in this study. First, the used coil shows a considerable gradient of B_1_+ in the anterior-posterior direction. This causes variations of the actual excitation flip angle between the anterior and posterior regions of the heart depending on the anatomy of specific subject. Based on estimations using low flip angle gradient echo sequences, at least a variation by a factor of 2 has to be expected. Relative B_1_ variations depend on the distance between the anterior and posterior section of the coil and, thus, on the study subject’s weight, sex and thorax anatomy (fat and muscles distribution). The currently implemented by vendor B_1_ -mapping procedure does not acquire the B_1_-maps needed for the flip-angle calibration in a cardiac triggered mode within adequate breath-hold time (>1 min is needed), thus is not feasible for CMR. Second, the largest obtainable flip angle is limited by SAR limits which in practice limit the number of excitation pulses per heartbeat. All of these factors, together with the necessity to reach a sufficient excitation on the posterior region of the heart and to optimize the blood-tissue contrast required these individual adjustments of the flip angle according to the specific measurement situation. Our data also show that the nominal flip angle influences the image quality and interpretability of the right ventricle. This observation is most likely attributable to the closer proximity of the right ventricle to the anterior part of the RF coil which might also account for the significance levels detected for the influence of the coil positioning on the right ventricular image quality.

Another aspect in the comprehensive approach towards a routinely used CMR at 7T is the subjective acceptance of the examination by the volunteer. The results of several larger studies demonstrate the overall good acceptance and lack of severe side effects of 7T MRI in general [35,36]. The reported other side effects in our study, especially vertigo during the table movement, fall in line with previously reported studies and further support the good acceptance of 7T MRI [35,36,51,52].

In our study a limiting factor was that due to the exploratory character, no well- defined clinical measurement protocol was followed, affecting the CINE imaging in particular. For the technical character of this manuscript, we think this is an acceptable drawback. On a small scale, a comparison between CMR data at 7T and at clinical field strengths showed a good agreement, however, a further one-to-one comparison of 7T with 3T clinical data in larger numbers, as well as comparative studies in patients with cardiac disease will be required to validate the functional assessment of CMR at 7T [20]. The RF coil is crucial for patient safety and optimal image quality. Therefore, it will be interesting to observe in future studies how promising new coil concepts which have been tested successfully in large [53,54] and in other pilot studies [55–58] will further improve the image quality in clinical imaging. Additionally, the use of automated segmentation methods based on transfer learning might add to the application of CMR at 7T [59,60].

Overall, the results of our study covering information on 84 subsequent CMR examinations at 7T MRI demonstrates that already at this time cardiac applications with commercially available setups allow for a solid morphological and functional analysis as a foundation for future clinically orientated studies. on cardiac morphology and function in patients using 7T MRI. Diagnostic cardiac image data, as well as flow measurements in the ascending aorta can be obtained with already existing techniques and pulse sequences. This data encourages to progress towards routine CMR in patients but also highlights the need to further develop advanced 7T technology.
